# Therapeutic ketogenic diet as treatment for anorexia nervosa

**DOI:** 10.3389/fnut.2024.1392135

**Published:** 2024-09-04

**Authors:** Guido K. W. Frank, Barbara Scolnick

**Affiliations:** ^1^Department of Psychiatry, San Diego School of Medicine, University of California, San Diego, San Diego, CA, United States; ^2^Boston University, Boston, MA, United States

**Keywords:** anorexia nervosa, metabolism, ketogenic, brain, behavior, treatment

## Abstract

Anorexia nervosa (AN) is a severe psychiatric disorder. However, we lack neurobiological models and interventions to explain and treat the core characteristics of food restriction, feeling fat, and body size overestimation. Research has made progress in understanding brain function involved in the pathophysiology of AN, but translating those results into biological therapies has been challenging. Studies have suggested that metabolic factors could contribute to developing and maintaining AN pathophysiology. Here, we describe a neurobiological model for why using a therapeutic ketogenic diet could address key alterations in brain function in AN and prevent the desire for weight loss and associated eating disorder-specific symptoms. This translational model is based on animal studies and human data and integrates behavioral traits, brain neural energy metabolism, and neurotransmitter function. Pilot data indicate that the intervention can dramatically reduce eating and body-related fears, although larger studies across illness stages still need to be conducted.

## Introduction

Anorexia nervosa (AN) is a severe psychiatric illness characterized by food avoidance, severe emaciation, and a perception of being overweight despite a very low body weight (1). AN is a chronic disorder with frequent relapse, high disease burden, and treatment cost (2–6). Treatment effectiveness, however, is limited (7, 8). AN has a mortality rate twelve times higher than the death rate from all causes of death for females 15–24 years old (5, 6, 9). AN shows a complex interplay between neurobiological, psychological, and environmental factors, and little is known about the pathophysiology or biomarkers that characterize AN when underweight or weight recovered (10, 11). Notably, in individuals with AN after weight recovery (wrAN), fears of weight gain, body dissatisfaction, and body image distortion are often elevated similarly or even higher compared to the underweight state, can persist for many years, and pose a risk for renewed self-starvation and relapse (4–6, 12–15).

Brain research before and after weight restoration has indicated alterations in circuits that compute reward valence or motivational salience of stimuli (stimuli that propel an individual’s behavior toward or away from a particular stimulus), which, together with a conditioned fear of weight gain, may contribute to the vicious cycle of self-starvation (2, 16, 17). The neural basis of what drives self-starvation remains poorly understood (18). A better mechanistic understanding of what triggers and perpetuates core behaviors such as food restriction neurobiologically in AN and wrAN, besides an environmentally driven ideal of thinness, would help develop more effective treatments (11). However, important aspects of the AN pathophysiology are still largely not yet identified, and there is a lack of biological treatments available for AN when underweight, or relapse prevention after weight restoration, despite decades of brain research. In this hypothesis and theory article, we propose a neurobiological model based on animal studies and human pilot data and integrates behavioral traits, brain neural energy metabolism, and neurotransmitter function, supporting a therapeutic ketogenic diet in AN.

## Neurobiology of AN

Brain research from various groups over the past decades has provided empirical data to better understand symptoms and behaviors in AN. Studies have found changes in the neurotransmitter systems for serotonin and dopamine in AN (8), altered brain structure, and more recently, tasks that engaged specific brain circuits that separated AN from healthy control groups (19–22). Those latter studies found evidence that AN is associated with altered brain function for processes involving the reward circuitry, cognition, emotion-regulating pathways, and regions that process executive function (21, 23, 24). Studies from our group, for instance, suggested that food avoidance and weight loss in AN alter circuits that process motivational salience (what stimuli to approach or avoid) and reward valence (whether expectations are violated) and involve cortical and subcortical regions that affect appetitive drive (16, 19, 25–33).

The primary brain regions that compute motivational salience and reward valence include the ventral striatum, insula, and orbitofrontal cortex (34). Another region, the amygdala, acts as an “early integrating” brain region for the salience of stimuli. It responds to expectation and triggers the dopamine response and associated behaviors in the nucleus accumbens and ventral striatum (35–37). Importantly, the frontal cortex processes whether an individual feels safe or whether a situation is associated with threat and fear, which activates those dopaminergic circuits to drive either approach or dread and avoidance (38, 39). Those mechanisms apply to AN, where fear of weight gain causes food and eating to become conditioned fear-inducing stimuli, leading to negative ruminations and ambiance (40, 41). The amygdala, in turn, responds to those fear and anxiety-inducing stimuli, modulates the dopaminergic motivational salience response, and triggers food avoidance (36, 42–44).

Individuals with AN overestimate their body size even when they are thin or underweight. Environmental factors, including thin-body messages from the media, may trigger those thoughts and condition a fear response (45–47). A “multisensory impairment of body perception” was proposed in some studies (48–50), but others suggested that affective factors drive body size overestimation in AN instead (50–53). Interestingly, some studies raised the possibility of a frontal cortical cognitive dysfunction in AN (54), and especially in restricting type AN, a psychotic-delusional component to the overvalued ideas of thinness could drive body size overestimation (55, 56). Those overvalued ideas may be driven by diminished sensory processing, which has also been associated with altered dopamine reward prediction error response (57). Brain dopamine activity is tied to glucose utilization, linking brain energy metabolism to a likely central aspect of AN neurobiology (57–59).

## Anxious traits and stress may affect brain metabolism in AN

Negative affect and deficits in regulating emotions, together with elevated anxious traits, are considered important for the etiology of AN (60–62). Unpleasant feelings such as anxiety, sadness, fear, or anger contribute to the negative affect experience and may drive AN behaviors (63). Worry, for instance, was related to fasting and fear of gaining weight or becoming fat in a sample of individuals with AN or wrAN (64). Stress and resulting negative affect may thus drive negative body image and body size overestimation (65, 66), consistent with ecological momentary assessment research showing that negative affect is involved in maintaining restrictive eating across AN subtypes (67–69). In wrAN, negative affect assessed over 2 weeks was related to a self-perception of inefficiency (70). Fear predicted increased dietary restraint, whereas dietary restraint predicted increased guilt and hostility (71). The importance of negative affect has been recognized already in youth with AN, and negative affect may contribute to developing AN (41, 72). Furthermore, difficulties with negative affect and emotion regulation persist long into recovery and may pose significant long-term risk factors for relapse (73).

It has long been known that stress affects glucose metabolism, altering blood sugar levels (74). Recent research has refined those studies. For instance, animal studies found that 40% of mice had a stress-susceptible phenotype associated with elevated blood glucose but reduced brain glucose metabolism, suggesting a specific mechanism in susceptible individuals (75). It was subsequently hypothesized that stress-related disorders could be associated with altered glucose metabolism (76). Research in humans indicated, for instance, altered brain glucose metabolism after acute stress using the Trier Social Stress Test (77), or in individuals with a chronic stress condition, posttraumatic stress disorder, showing lower brain glucose metabolism after administration of the stress hormone hydrocortisone (78). A recent study in cancer patients showed that negative affect was negatively correlated with glucose metabolism across cortical and subcortical brain regions, indicating global effects (79).

Those studies may have direct implications for the pathophysiology of AN or wrAN as self-perceived stress and sensitivity to negative affect may create a condition of chronic stress affecting brain glucose metabolism. This hypothesis is supported by studies that showed elevated baseline levels of the stress hormone cortisol in AN, altered cortisol response to a stressor compared to controls, and altered stress axis response that persisted in wrAN (80). That research directly associated stress and “psychological burden” with the biological stress response in AN, including stress axis function.

It has previously been hypothesized that the pathophysiology of AN may include metabolic abnormalities, or AN may be a “metabolic disorder of psychological origin” (81), which has only recently again received attention (82, 83). We propose, based on the above-described literature, that elevated anxious traits and distress over negative affect in individuals prone to AN interfere with brain glucose utilization and, thus, normal brain metabolism and function (84, 85).

## AN as a metabolic disorder

Several studies have found metabolic abnormalities in AN (81, 82) and associated elevated oxidative stress and inflammatory markers (86, 87). Recent genetic studies suggested that AN is associated with metabolic traits as a possible risk factor for developing the condition (88). A model that integrated biological and environmental factors hypothesized that anxiety and stress are critical factors in AN that drive altered glucose distribution between brain and body and induce changes in the lateral hypothalamus-pituitary–adrenal axis (89). That model is consistent with behavioral studies that indicated elevated intolerance of uncertainty and anxiety in AN, triggering the body’s biological stress response (90, 91). Stress leads to an increased allocation of glucose to the brain; however, while stress is associated with higher glucose *needs*, it is also associated with decreased glucose brain *utilization*, particularly in frontal cortical, thalamic, hippocampal, and temporal regions (85, 92, 93). In some individuals, this increased glucose requirement may deplete body energy resources; in others, reduced glucose utilization in the context of stress may lead to overeating (89, 90, 94). This model has been hypothesized to be relevant for psychiatric disorders, and computational models support the underlying premise (95, 96).

Research on the metabolic underpinnings of AN is still largely lacking. An animal study that evaluated glucose metabolism in nine female rats modeled AN brain response and randomized the animals to food restriction or regular food access (97). That study suggested *increased* glucose metabolism in the cerebellum but *decreased* metabolism in the hippocampus and striatum after food restriction. A few genetic studies suggested altered energy, including glucose homeostasis in AN (83, 98–101), and AN has been associated with altered glucose metabolism related to changes in insulin sensitivity (99). Others found mitochondrial dysfunction in AN related to oxidative stress and altered metabolic signaling (102), or metabolic dysfunction in the gut microbiome in AN that was opposite to that found in high-weight individuals (83). Those data, together with genetic markers, supported the possibility of critical metabolic targets that need to be identified for successful treatment development for AN (83). Only a few human brain imaging studies have investigated glucose metabolism in AN compared to healthy controls, and the samples were generally small and results inconsistent. One earlier study found glucose *hypometabolism* in AN (*n* = 10) that tended to normalize with weight gain (103), while another found glucose *hypermetabolism* in a small sample (*n* = 5) in cortical and subcortical regions (104). Another study in underweight AN (*n* = 14) found *lower* regional glucose metabolism in the anterior and posterior cingulate, dorsolateral prefrontal cortex, left middle temporal, and right superior temporal gyrus (105). After hormone replacement in six individuals with AN, regional glucose metabolism normalized in the anterior and posterior cingulate, premotor, parietal cortex, and caudate nucleus. A study that investigated brain glucose metabolism in AN before and after nucleus accumbens brain stimulation found *increased* regional glucose metabolism in AN (*n* = 6) versus healthy controls (*n* = 12) in the bilateral superior, medial and inferior frontal cortex, bilateral amygdala and hippocampus, left insula and bilateral putamen (106). The authors further reported that frontal cortical and hippocampal hypermetabolism *decreased* with nucleus accumbens stimulation.

Overall, the human neuroimaging results are mixed. The studies, in part, are decades old, and potential confounds such as nutritional status or comorbidity were not considered. Glucose metabolism has been linked to the neurotransmitters dopamine and serotonin, and altered neurotransmitter function found in AN as described above further supports the hypothesis of altered energy homeostasis, including glucose metabolism in AN (107, 108).

**Figure 1:**
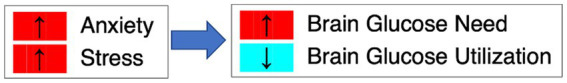
Anxiety, stress and brain glucose metabolism. We hypothesize that in AN, high anxiety and stress lead to elevated brain glucose need but reduced glucose utilization.

We propose that in individuals who have a predisposition for AN, there is reduced utilization of glucose in the brain despite high needs due to high anxious traits and sensitivity to stress and negative affect, which interfere with brain glucose metabolism (Figure 1) (91, 109–114).

While there is evidence that the vicious cycle of weight loss followed by more food restriction is, at least in part, due to the interactions between conditioned fear of weight gain and altered dopamine circuit function, which may drive dread and avoidance (16), we propose that there are fundamental metabolic abnormalities in individuals who develop AN that drive the development of the illness, hinder recovery from AN and trigger relapse in wrAN. Individuals with AN often report that food restriction reduces anxiety and improves mood (115). This may be due to cognitive and emotional factors where weight loss is seen as a success toward a certain body shape or in part due to elevated cortisol release, although the results are mixed (116–119). Here, we postulate that individuals with AN may have a *metabolic reason* why it is so desirable for them to pursue the starvation state. Individuals who develop AN tend to score high on state and trait anxiety and have perfectionistic traits that drive fear of failure and anxiety (28, 42, 120–124).

We hypothesize that high state and trait anxiety levels create ongoing interference with brain glucose utilization in AN as a risk factor before, during, or after weight loss. If a person with that disposition loses weight and enters a ketosis state, the brain will use ketones as an alternative energy source that may be less affected by anxiety. Thus, the individual learns that starvation paradoxically provides a better subjective feeling of having sufficient energy, and food restriction becomes self-reinforcing. However, this state also depletes the body’s resources and eventually leads to death. We propose that providing a person with that disposition with ketone bodies while ensuring normal weight will remove the desire to self-starve and support weight maintenance.

## The therapeutic ketogenic diet provides an alternative energy source to reduce anxiety and normalize inflammation

TKD is beneficial in neurological conditions such as seizure disorders, and there is emerging evidence that it might also be an effective intervention to treat psychiatric conditions (125–127).

TKD mimics some aspects of fasting (128). During fasting, the body faces an energy deficit in its cellular fuel supply as glucose and insulin levels decrease. The metabolism of white fat increases, and the resulting fatty acids are used to supplement the energy needs of most organs. The brain is unique, however, because fatty acids cannot easily cross the protective blood–brain barrier (129), and thus, the brain is highly sensitive to drops in glucose. For decades, it was thought the brain could only utilize glucose (130). This presented an enigma because human glucose stores are limited and can be sustained for only a few days. Yet, there have been many documented instances where individuals are capable of fasting (as long as they drink water) for several weeks before succumbing. This paradox was explained in an experiment performed on three obese patients who were starved for 6 weeks in a metabolic ward, and their internal carotid arteries and jugular veins were cannulated at the start and end of the experiment (131). The study demonstrated that beta-hydroxybutyrate and acetoacetate replaced glucose as the predominant fuel for brain metabolism.

The underlying principle of TKD is that most energy is supplied via fat in the diet, which is then broken down into fatty acids for energy consumption (132). However, sufficient calories are in the food to maintain normal weight. During fasting or TKD, fat metabolism increases, and fatty acids are transported to the liver. Fatty acids are composed of long chains of carbons. In the liver, fatty acids are ordinarily converted into acetyl-CoA, which enters the tricarboxylic acid (TCA) cycle. When fatty acid levels are elevated and exceed the metabolic capacity of the TCA cycle, acetyl-CoA is shunted to ketogenesis. Two acetyl-CoAs can combine through a thiolase enzyme to produce acetoacetyl-CoA, a precursor for acetoacetate synthesis (ACA) and β-hydroxybutyrate (BHB). Acetone, the other major ketone body, is produced primarily from spontaneous decarboxylation of ACA and can be eliminated as a volatile substrate through the lungs and kidneys. In the blood, ACA and BHB are transported from the vascular lumen to the brain interstitial space and both glia and neurons by monocarboxylic acid transporters (MCTs). MCT-1 is the principal carrier localized to the vascular endothelium. Within neurons, both ACA and BHB are transported directly into mitochondria and then converted to acetyl-CoA through several enzymatic steps. BHB is converted to ACA through D-β-hydroxybutyrate dehydrogenase, and ACA undergoes subsequent conversion to acetoacetyl-CoA through a succinyl-CoA transferase enzyme. Finally, acetoacetyl-CoA-thiolase converts acetoacetyl-CoA to two acetyl-CoA moieties, which then enter the TCA cycle (132).

TKD or exogenous ketones have been associated with marked changes in brain glucose metabolism. Specifically, elevated blood ketone levels resulted in lower brain glucose uptake in humans, which was studied using the radiotracer [^18^F]fluorodeoxyglucose ([^18^F]FDG) and positron emission tomography (PET). In one study, infusion of BHB ketone bodies reduced brain glucose uptake and enhanced blood flow, supporting the notion of TKD’s neuroprotective effects (133). A study that briefly applied a ketogenic diet found regionally specific effects of blood ketosis on lowering brain glucose uptake (134), including the precuneus, a brain region necessary for visuospatial function, episodic memory retrieval, and self-referential processing, affecting one’s perceptual image or mental concept of oneself (135), which could have implications for AN in the pathophysiology of body image distortion (136, 137). The ketogenic diet in that study was maintained only for 48 h, and results may differ after prolonged ketosis as in this study (134). A study testing the effects of ketogenic diet over 4 days led to global decreases in glucose metabolism across widespread cortical and subcortical regions, with the strongest decrease in the middle frontal gyrus (Brodmann area 8, 46) followed by the frontal pole (Brodmann area 10) and cuneus (Brodmann area 17) (138). Three weeks of a ketogenic diet in an animal model also led to widespread cortical reductions in glucose metabolism (139). Thus, short and longer-term ketogenic diets led to extensive regional glucose metabolism reductions, with longer duration associated with more extensive glucose metabolism decreases. The above-referenced study by Courchesne-Loyer et al. (138) suggests that the middle frontal cortex is most affected. However, there were large reductions across all frontal, temporal, parietal, occipital, cingulate, and subcortical regions tested. Those data indicate large global decreases without specific circuits delineated, although some areas were more affected than others (138).

The metabolic shift with TKD is associated with a variety of central nervous system and general effects on the body. Aside from the ketone bodies enhancing cell energy metabolism by replenishing the metabolic pathway, TKD has been associated with reducing oxidative stress and inflammatory processes and regulating neurotransmitter systems (140–142), which are all processes implicated in the pathophysiology of AN (21, 23, 86, 87). Furthermore, replacing glucose with ketone bodies via TKD to supply the brain with energy enhances γ-aminobutyric acid (GABA) in the brain via enhanced glutamate production converted to glutamine and GABA (143). GABA is a primary inhibitory neurotransmitter that reduces anxiety (144, 145). In the animal model, enhancing systemic ketone body levels reduced stress and anxiety (146, 147). In AN, altered GABA function has been reported in an animal model for AN, and enhancing GABA via ketosis might effectively reduce AN-specific and non-specific anxiety (148, 149). Other studies have found elevated inflammatory markers in AN, and elevating blood ketone levels has been shown to reduce inflammation (150).

## Metabolism as neurobiological target in AN

It has been hypothesized that brain metabolic alterations, perhaps relating to cell mitochondria, have a critical role in psychiatric disorders (151, 152). Psychiatric disorders have been associated with inborn errors of metabolism, supporting the link between altered metabolism and psychiatric pathophysiology (153). Research has also increasingly recognized other abnormalities associated with psychiatric conditions, such as elevated inflammatory markers and markers for oxidative stress (154–156). It has been hypothesized that AN’s pathophysiology includes metabolic abnormalities (82), or that AN may be a “metabolic disorder of psychological origin” (81). Thus, there is growing evidence that nutrition and mental health are linked, and diet may be particularly appealing as a therapeutic intervention (157).

TKD has received increasing attention since it was found effective in pediatric epilepsy (158). Others have suggested that TKD could improve autism-related behaviors, symptoms associated with Alzheimer’s disease, or disorders related to mood or psychotic symptoms (159–161). These findings have led to the suggestion that TKD could be an effective metabolism-directed intervention for psychiatric conditions (162, 163).

## A neurobiological model for TKD as a possible treatment intervention for AN

**Figure 2:**
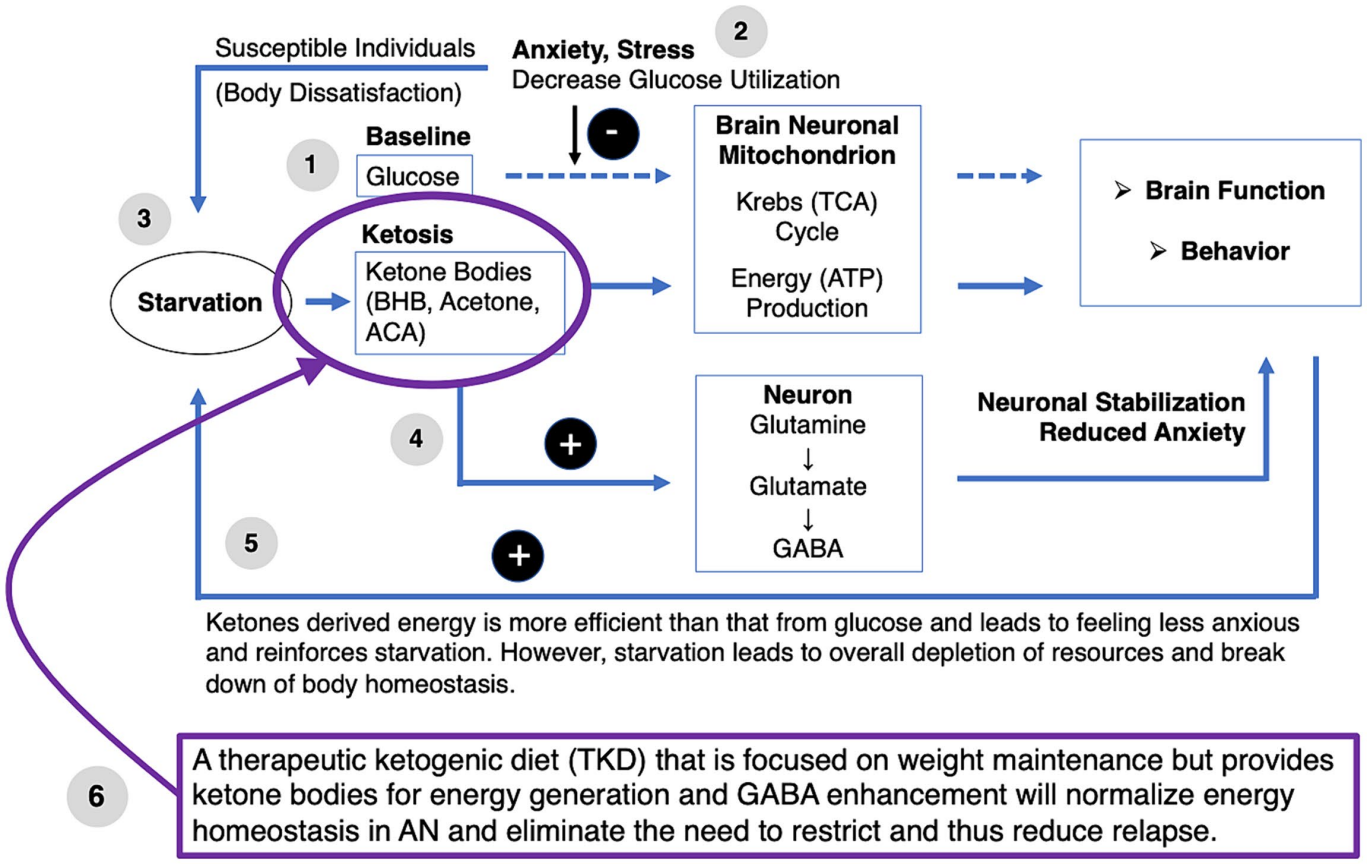
Therapeutic ketogenic diet in anorexie nervosa conceptual model. ACA, acetoacetate; BHB, beta-hydroxybutyrate; TCA, tricarboxylic acid cycle.

The TKD may be an effective treatment intervention for AN to normalize energy homeostasis and remove the need to self-starve for nutritional ketosis. Figure 2 provides a conceptual model of dysfunctional brain glucose metabolism and the therapeutic effects of TKD (85, 93, 132, 147, 164). (1) At baseline and under typical conditions, glucose is used by the brain mitochondria to generate energy and support brain function and associated behaviors. (2) In individuals with AN, high levels of anxiety and perfectionism lead to stress, which reduces glucose utilization despite high energy needs. (3) Body dissatisfaction and drive for thinness in susceptible individuals drive starvation. Transient imbalances between nutritional intake and energy requirements lead to the generation of ketones and the use of beta-hydroxybutyrate (BHB), acetone, and acetoacetate (ACA) as alternative energy compounds that enter the brain mitochondrial Krebs cycle and are better utilized than glucose and independent from the effects of stress. (4) Ketosis leads to elevated production of neuronal GABA via glutamine and glutamate, which may help with emotion regulation and reduce anxiety. (5) Ketosis leads to improved brain energy supply and elevated GABA production, which stabilizes neuronal function and causes positive feedback to promote further starvation-mediated ketosis. (6) A ketogenic diet that is energy-rich to accomplish weight maintenance in wrAN or weight gain in AN eliminates the need for ketosis via starvation, thus replacing “starvation ketosis” with “nutritional ketosis” (85, 93, 132, 147, 164).

## Supporting preliminary studies

**Figure 3:**
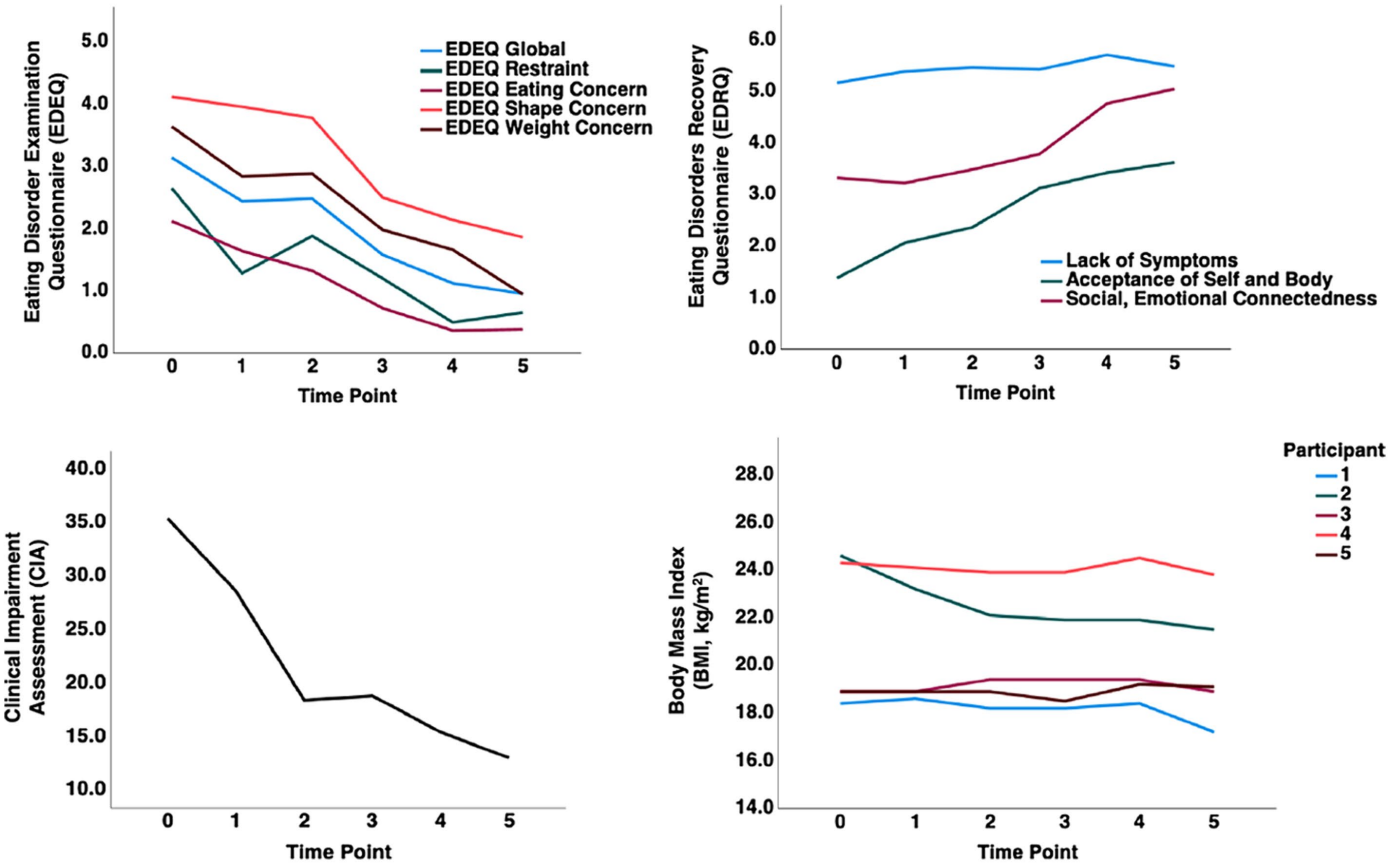
A pilot study showing therapeutic ketogenic diet and ketamine infusion effects in the treatment of chronic persistent eating disorder psychopathology in anorexia nervosa [Calabrese et al. (166), with permission]. Graphical representation of composite values for behavioral measures and body mass index (BMI) over time. Timepoint (TP) 0: Baseline, TP 1: 4 weeks TKD all subjects; TP 2: 8 weeks TKD (subjects 3, 4) or 4 weeks TKD + 4 weeks ketamine (subjects 1,2,5); TP 3: 1-month post-intervention; TP 4: 3 months post-intervention; TP 5: 6 months post-intervention.

A single case study suggested that TKD, followed by ketamine infusion, could help that patient recover (165). That individual remains recovered to this date (Dr. Scolnick, personal communications). That study laid the foundation for an IRB-approved protocol in five wrAN who were still highly affected by the illness (166). In that study, we conducted an open-label trial to test whether the case report response could be replicated. Those five wrAN adults with persistent eating disorder thoughts and behaviors adopted the TKD to maintain weight. In addition, participants received six ketamine infusions after 4 to 8 weeks of stable ketosis and were followed over six months. All participants completed the study protocol without significant adverse effects. The participants consumed the TKD for at least 8 weeks (4 to 8 weeks TKD alone, then with added ketamine for 4 weeks); two individuals continued TKD after the formal study intervention for a total of 4 months on TKD and two individuals for 6 months of TKD, suggesting good tolerability. The group showed significant improvements (repeated measures ANOVA) on the Clinical Impairment Assessment (*p* = 0.008), Eating Disorder Examination Questionnaire (EDEQ) Global score (*p* = 0.006), EDEQ-Eating Concerns (*p* = 0.005), EDEQ-Shape Concerns (*p* = 0.016), EDEQ-Weight Concerns (*p* = 0.032), Eating Disorders Recovery Questionnaire (EDRQ) Acceptance of Self and Body (0.027) and EDRQ-Social and Emotional Connection (*p* = 0.001). Weight remained stable during the trial. Figure 3 shows a change in composite scores and BMI over time. The baseline was at “0”; time point one indicates 4 weeks of TKD for all participants, timepoint 2 indicates 8 weeks of TKD for two subjects, and 4 weeks of TKD plus ketamine in 3 subjects; time points three and later are post study intervention assessments. EDEQ global score, Restraint, Eating Concern, Weight Concern, Acceptance of Self and Body, and Clinical Impairment showed steep improvements before adding ketamine, suggesting that TKD alone was highly effective.

Of note, in the case report and the case series (165, 166), ketamine infusions were added once the subjects had been on the TKD for at least 4 weeks. The choice was clinically driven and based on a small positive report of decreased obsessions/compulsions in a pilot study of twelve patients with AN (167). Ketamine is an N-methyl D-aspartate (NMDA) glutamate antagonist that has been in use since the 1960s as an anesthetic (168). Over the past two decades, ketamine has been in use as a rapid antidepressant agent (169). There have been accelerating efforts to discern the mechanism of action and focus on the effects of ketamine on energy metabolism, mitochondrial function, and glutamate/GABA function (170, 171). All these areas overlap with the effects of TKD, which corresponded to our clinical observation that the two modalities (TKD and ketamine) led to extended improvement. This warrants further study.

Four of the 5 study participants have remained recovered (low symptom scores, normal weight) for at least 12 months since the end of the study; one participant who stopped TKD after 8 weeks relapsed 4 months after treatment (unpublished data). The small study suggested that this novel treatment is safe and effective for wrAN adults with chronic AN-related psychopathology. The results from this open trial supported the idea that specific neurobiological underpinnings for AN can be modified with TKD.

## Discussion

AN remains a severe psychiatric disorder without approved biological intervention. The above neurobiological model, with evidence from basic science and human genetic and preliminary clinical data, supports the possibility that brain metabolism may be a key target for intervention to treat this disorder and provide a treatment that targets the disorder’s pathophysiology mechanistically. The pilot data to date are from weight recovered individuals. We are currently conducting a follow-up study in a larger group of individuals in the wrAN group to test for reduction of thoughts, feelings, and behaviors that are specific to AN. Future studies will need to investigate individuals underweight with AN when we have further indications that this treatment is safe and effective. An important aspect to also consider is that while we can change neurobiology and provide effective treatment, individuals with AN have often learned to live with the disorder. Once those thoughts, feelings, and behaviors are diminishing, the individual has to re-organize their life, which can be anxiety-provoking in itself. In summary, there is much reason to believe that a TKD could support treatment outcomes in AN, and further study is needed to understand the underlying mechanisms *in vivo* and in relation to specific illness behaviors.
